# Dual effect of radiotherapy related concomitant cardiovascular diseases in non‐small cell lung cancer

**DOI:** 10.1002/cam4.4948

**Published:** 2022-06-26

**Authors:** You Mo, Baoqing Tian, Meng Wu, Minxin Chen, Dawei Chen, Jinming Yu

**Affiliations:** ^1^ Shantou University Medical College Shantou China; ^2^ Department of Radiation Oncology Shandong Cancer Hospital and Institute, Shandong First Medical University and Shandong Academy of Medical Sciences Jinan China

**Keywords:** cardiac deaths, epidemiology, mortality, non‐small cell lung cancer, radiation therapy

## Abstract

**Background:**

Nowadays, cancer and cardiac diseases are two of the most causes of death, so cancer treatment‐related cardiac death cannot be ignored. For lung cancer, chest radiotherapy (RT) is essential, but the related cardiotoxicity has not been fully studied.

**Methods:**

We reviewed the data of 11,455 patients with non‐small cell lung cancer (NSCLC) from the Surveillance, Epidemiology, and End Results database from 2001 to 2015. The change trend for concomitant cardiovascular diseases (CVD)‐specific death was calculated and graphically demonstrated. Univariate and multivariate analyses for survival were performed using Cox risk regression model.

**Results:**

In our analysis, the overall incidence and mortality from NSCLC declined, but CVD‐specific death increased. Both chemoradiotherapy and radiotherapy alone played a significant role in CVD‐specific death. Analyzed longitudinally from diagnosis, we found that the effect of RT in CVD‐specific death increased continuously over the third years and the hazard ratio for CVD‐specific death was 1.386 times between RT and non‐RT group (HR = 1.386, 95% CI 1.322–1.452; *p* < 0.0001). On the other hand, RT played a protective role in CVD‐specific death before the second years, especially in recent years from 2013 to 2015 (HR = 0.843, 95% CI 0.740–0.959; *p =* 0.009).

**Conclusions:**

Although the mortality from NSCLC decreased, but radiotherapy‐related CVD‐specific mortality cannot be ignored. In the long‐term over 3 years, RT significantly promoted CVD‐specific death. However, RT turned to be a protective role in the short‐term within 2 years. In clinical practice, we need to comprehensively consider the dual effects of radiotherapy on the side effect of heart.

## Graphical abstract

The overall incidence and mortality from non‐small cell lung cancer declined, but cardiovascular diseases (CVD)‐specific death increased. In the long‐term over 3 years, radiotherapy (RT) significantly promoted CVD‐specific death. However, RT turned to be a protective role in a short‐term within 2 years.

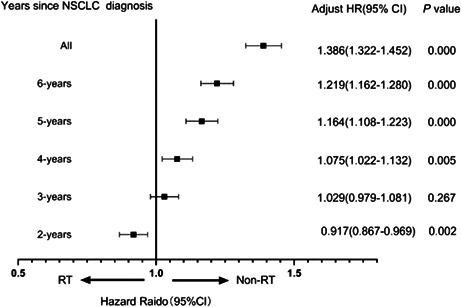

## 1 INTRODUCTION

Cancer and cardiac diseases are two of the leading causes of deaths worldwide. And Lung cancer is the second most common malignancy with highest mortality.^1^ The improvement of cancer therapy is effective in prolonging patients' survival. And the growing population of survivors may develop short‐term and long‐term side effects of cancer therapy. So cancer treatment‐related cardiac death cannot be ignored.

Since the early 1900s, ionizing radiation has remained a major modality in cancer treatment. In one lung cancer screening program, 31% of patients received curative radiotherapy.^2^ To date, radiotherapy (RT) played an essential role in patients with non‐small cell lung cancer (NSCLC). Advances in radiation oncology have made the radiation delivery more precise while reducing the radiation dose to the surrounding normal tissue, but the heart injury from radiation remains a problem. Cardiac radiation dose exposure is a modifiable cardiac risk factor for major adverse cardiac events (MACE) in patients with locally advanced NSCLC.^2^ Meanwhile, major improvements have been made in NSCLC treatment with the advent of targeted therapies and immunotherapies. In addition, but combination therapy may give rise to more toxicity and inability to avoid it. Along with an increase in the number of cancer survivors, the cardiotoxicity spectrum of cancer therapies has changed, therefore cardiotoxicity remains a great concern.

Studies on the causes of death among patients with NSCLC have revealed that concomitant cardiovascular diseases (CVD) present a formidable health problem. A previous study reported that heart diseases accounted for approximately 5.3% of the total deaths in stages I–III NSCLC, only secondary to the primary disease.^3^ Results from a study of hospitalized patients with cancer have revealed that the prevalence of hypertension in lung cancer was 24.5%, and the majority of patients were at a high level and very‐high level of cardiovascular risk.^4^ Although few patients were diagnosed with CVD before treatment. Previous studies suggested that immunotherapy in patients with NSCLC demonstrates decreased overall survival (OS) in heart disease‐specific deaths.^5^ An increasing number of patients with comorbidities are likely to suffer from adverse effects of treatment. Nevertheless, evidence on the adverse effects of RT, including both short‐term and long‐term cardiotoxicity, remains limited. Thus, we aim to investigate the relationship between radiotherapy and CVD‐specific death and evaluate prognostic factors for CVD‐specific death among patients with NSCLC using the Surveillance, Epidemiology, and End Results (SEER) database. We focus on the short‐term and long‐term effect of RT for CVD‐specific death.

## 2 MATERIALS AND METHODS

### 2.1 Data

The National Cancer Institute's SEER database is an authoritative source of information on cancer incidence and survival in the United States; collected data contains patient demographics, clinicopathological features, treatment, and follow‐up for vital status. Our study population was chosen from the SEER database (SEER 18 Regs Custom data [with additional treatment field], Nov 2018 [1975–2016 varying]). The SEER program includes 18 population‐based tumor registries areas from 1975 to 2016. We used the SEER*Stat software (version 8.3.8) for data extraction (reference number 18157‐Nov 2019).

**FIGURE 1:**
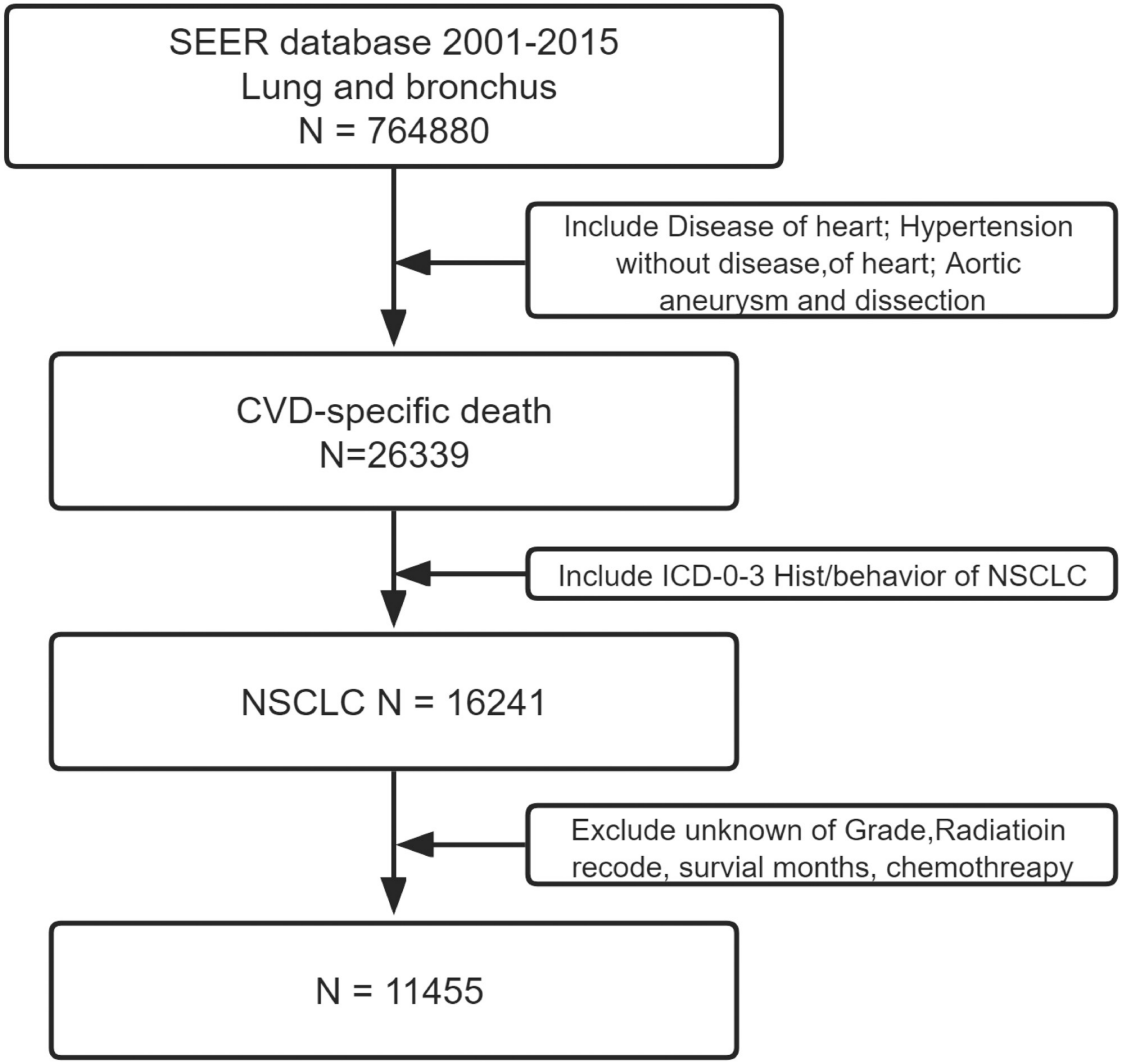
Patients selection diagram

We identified our patient population by querying “site recodes ICD‐O‐3/WHO 2008” with the term “lung and bronchus” as the primary site. For each case, we requested the following information: race (white, black, or other), age (≥18 years), year of diagnosis (2001–2015), sex (male or female), tumor laterality (right, left, or other), Grade (I–II, III–IV), radiotherapy (yes or no), chemotherapy (yes or no), ICD‐0‐3 his/behavior, Cause of Death (COD) to site record, survival months, and vital status. The inclusion criteria were: (i) Patients diagnosed from 2001 to 2015; (ii) COD to site record, SEER recodes the cause of death based on International Classification of Disease (ICD) 10th revision codes (available at URL: https://seer.cancer.gov/codrecode/1969_d04162012/). Patients were considered to have died of cardiovascular diseases when their SEER cause of death recodes was 50,060, 50,070 or 50,100. This corresponds with the following ICD 10th revision codes: I00 through I09, I10, I11, I12, I13, I20 through I51or I71. and (iii) NSCLC histological types, namely NSCLC, squamous and transitional cell (International Classification of Diseased for Oncology‐3 [ICD‐O‐3] codes 8051, 8052, 8070–8076, 8078, 8083, 8084, 8090, 8094, 8120 and 8123), adenocarcinoma (8015, 8050, 8140, 8141, 8143–8145, 8147, 8190, 8201, 8211, 8250–8255, 8260, 8290, 8310, 8320, 8323, 8333, 8401, 8440, 8470, 8471, 8480, 8481, 8490, 8503, 8507, 8550, 8570–8572, 8574, and 8576), large cell 8 (012–8014, 8021, 8034, and 8082), non‐small‐cell carcinoma, not otherwise specified (8046), and other specified carcinomas (8003, 8004, 8022, 8030, 8031–8033, 8035, 8200, 8240, 8241, 8243–8246, 8249, 8430, 8525, 8560, 8562 and 8575). The exclusion criteria were unknown grade, unknown radiotherapy, unknown chemotherapy, and missing the survival month. The workflow was illustrated in Figure 1. We also queried age‐standard mortality, NSCLC mortality, cancer‐specific mortality, and CVD‐specific mortality from the SEER database.

### 2.2 Ethics statement

All analyses were designed and performed in accordance with the ethical standards of the responsible committee on human experimentation and the Helsinki Declaration (1975, revised in 1983).

### 2.3 Statistical analysis

We used the Joinpoint software version 4.7.0.0 to characterize time trends in the age‐standardized rates (details are provided in the Appendix S1). Analyses were performed using SPSS Statistics for Windows version 25. Normally distributed data are expressed using mean and standard deviation, and data that were not normally distributed are expressed as medians by the quartering method. The characteristics of patients who did and did not receive radiotherapy were analyzed using Pearson's chi‐square statistics. We compared the differences in demographics, clinicopathology, and treatment, and divided patients according to the year of diagnosis (2001–2004, 2005–2008, 2009–2012, and 2013–2015) and explored CVD‐specific death chronologically, including 2, 3, 4, 5, and 6 years. Incidence, total mortality, cancer‐specific mortality, and cardiac‐specific mortality of patients with NSCLC were estimated by age adjustment in the SEER database. A cox proportional hazard model was used for univariate and multivariate analyses. For all analyses, *p* values <0.05 were considered significant.

## 3 RESULTS

### 3.1 CVD‐specific mortality is increasing continuously

All patients were enrolled based on The National Cancer Institute's SEER database from 2001 to 2005. The characteristics of patients who met the inclusion criteria were presented in Table 1. The study population comprised 11,455 patients as follows: 8173 patients (71.3%) who received RT and 3282 patients (28.7%) who did not receive RT. Here, 75.41% of patients received chemotherapy. Overall, age over 65 years (81.1%), white race or ethnicity (85.3%), and male (60.70%) predominance were high in all cases of CVD‐specific death in patients with NSCLC. Altogether, 50.4% of patients had Grade I or II lung cancer, while the others had Grade III or IV. A higher proportion of patients in the non‐RT group had adenocarcinoma, and similarly, those in the non‐RT group had squamous cell and transition cancers. Due to the duration of follow‐up, more patients were enrolled from 2001 to 2004, while the years 2012–2015 represented the lowest percentage.

**TABLE 1:** Patients characteristics

Characteristic	No. of patients (%)	RT	
		NO(%)	YES(%)
Race			
White	9768 (85.3)	7027 (86.0)	2741 (83.5)
Black	1202 (10.5)	806 (9.9)	396 (12.1)
Others	485 (4.2)	340 (4.2)	145 (4.4)
Sex			
Male	6956 (60.7)	4873 (59.6)	2083 (63.5)
Female	4499 (39.3)	3300 (40.4)	1199 (36.5)
Age, years			
<65	2164 (18.9)	1452 (17.8)	712 (21.7)
≥65	9291 (81.1)	6721 (82.2)	2570 (78.3)
Grade			
I and II	5777 (50.4)	4465 (54.6)	1312 (40.0)
III and IV	5678 (49.6)	3708 (45.4)	1970 (60.0)
Laterality			
Left	4983 (43.5)	3472 (42.5)	1511 (46.0)
Right	6367 (55.6)	4633 (56.7)	1734 (52.8)
Others	105 (0.9)	68 (0.8)	37 (1.1)
Tumor histology			
Squamous and transitional cell	4390 (38.3)	2968 (36.3)	1422 (43.3)
Adenocarcinoma	5202 (45.4)	4010 (49.1)	1192 (36.3)
Others	1863 (16.3)	1195 (14.6)	668 (20.4)
Chemothreapy			
No	8638 (75.41)	7009 (85.8)	1629 (49.6)
Yes	2817 (24.59)	1164 (14.2)	1653 (50.4)
Year of diagnosis			
2001–2004	4035 (35.2)	3003 (36.7)	1032 (31.4)
2005–2008	3534 (30.9)	2613 (32.0)	921 (28.1)
2009–2012	2710 (23.7)	1826 (22.3)	884 (26.9)
2013–2015	1176 (10.3)	731 (8.9)	445 (13.6)

**FIGURE 2:**
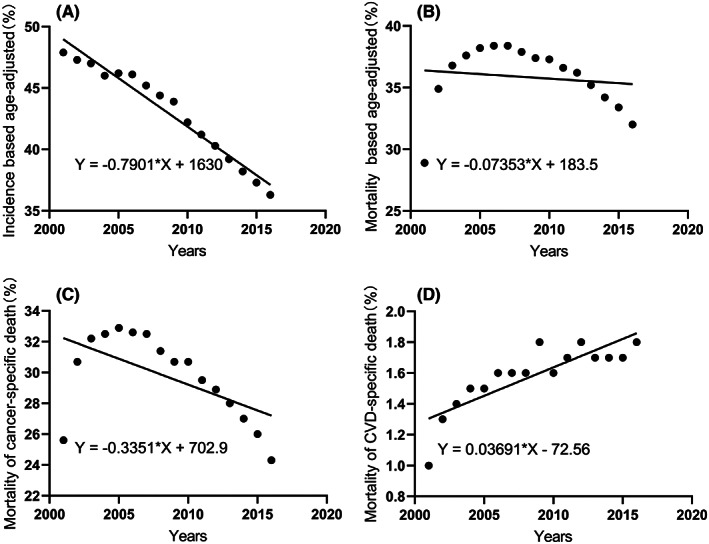
Incidence and mortality in patients with NSCLC. (A) Incidence; (B) Total mortality; (C) Mortality of cancer‐specific death; (D) Mortality of cardiac‐specific death

Figure 2A–C illustrated long‐term trends in morbidity, mortality, and cancer specific mortality in patients with NSCLC, they were all declining continually. However, the mortality for CVD‐specific death increased inversely (Figure 2D). The APC was shown in the Appendix S1. Mortality from NSCLC decreased over time (APC = −1.10), and the corresponding cancer‐specific mortality decreased by 2.02. Specifically, a rapid decline in mortality from NSCLC during the period from 2010 to 2015 was observed.

### 3.2 The long‐term effect of treatment for CVD‐specific death

**FIGURE 3:**
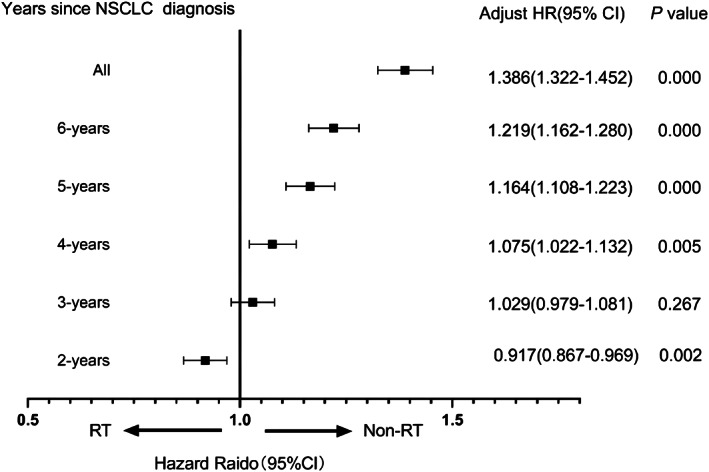
Hazard radio (95% CI) of RT in different years of of CVD‐specific death in patients with NSCLC

We wanted to explore what is causing the continued rise in CVD‐specific deaths. First, in the Cox regression hazard analysis, RT was found to be a significant predictor for CVD‐specific death in all patients. Considering the short‐term and long‐term effect of radiotherapy and chemotherapy on the heart, we explored CVD‐specific death chronologically, including 2, 3, 4, 5, and 6 years. Patients were subdivided into groups based on survival times. Meanwhile, our data suggest that RT was an independent factor for CVD‐specific death increased gradually over years (Figure 3). Multivariate analysis showed that RT was a protective factor in 2 years, while RT as a risk factor over 3 years of survival times. The difference between the RT and non‐RT was significant, with improvement in median survival of the RT group (HR = 0.917, 95% CI 0.867–0.969; *p* = 0.002) within 2 years. No significant difference was observed between the two groups for the third year (HR = 1.029, 95% CI 0.979–1.081; *p =* 0.267). In contrast, the non‐RT group was significantly better than the RT group for the fourth years (HR = 1.075, 95% CI 1.022–1.132; *p =* 0.005). RT was associated with an increase in the hazard ratio of CVD‐specific death from 1.164 to 1.386 for the fifth to sixth year (Figure 3). The results of the univariate and multivariate COX analyses were shown in the Supplementary Appendix (Appendix S2). There was no such trend for chemotherapy from the second to fifth year. Although the difference between the chemotherapy and non‐chemotherapy for NSCLC was significant in all patients (HR = 1.062, 95% CI 1.009–1.118; *p* = 0.022) (Appendix S3). The result suggested that long‐term adverse effects of both radiotherapy and chemotherapy were associated with CVD‐specific death over 6 years. In addition, black patients (HR = 1.161, 95% CI 1.088–1.239) and poorly differentiated grade (HR = 1.156, 95% CI 1.110–1.204) may have an increased risk for the CVD‐specific death.

### 3.3 The short‐term effect of RT for CVD‐specific death

**FIGURE 4:**
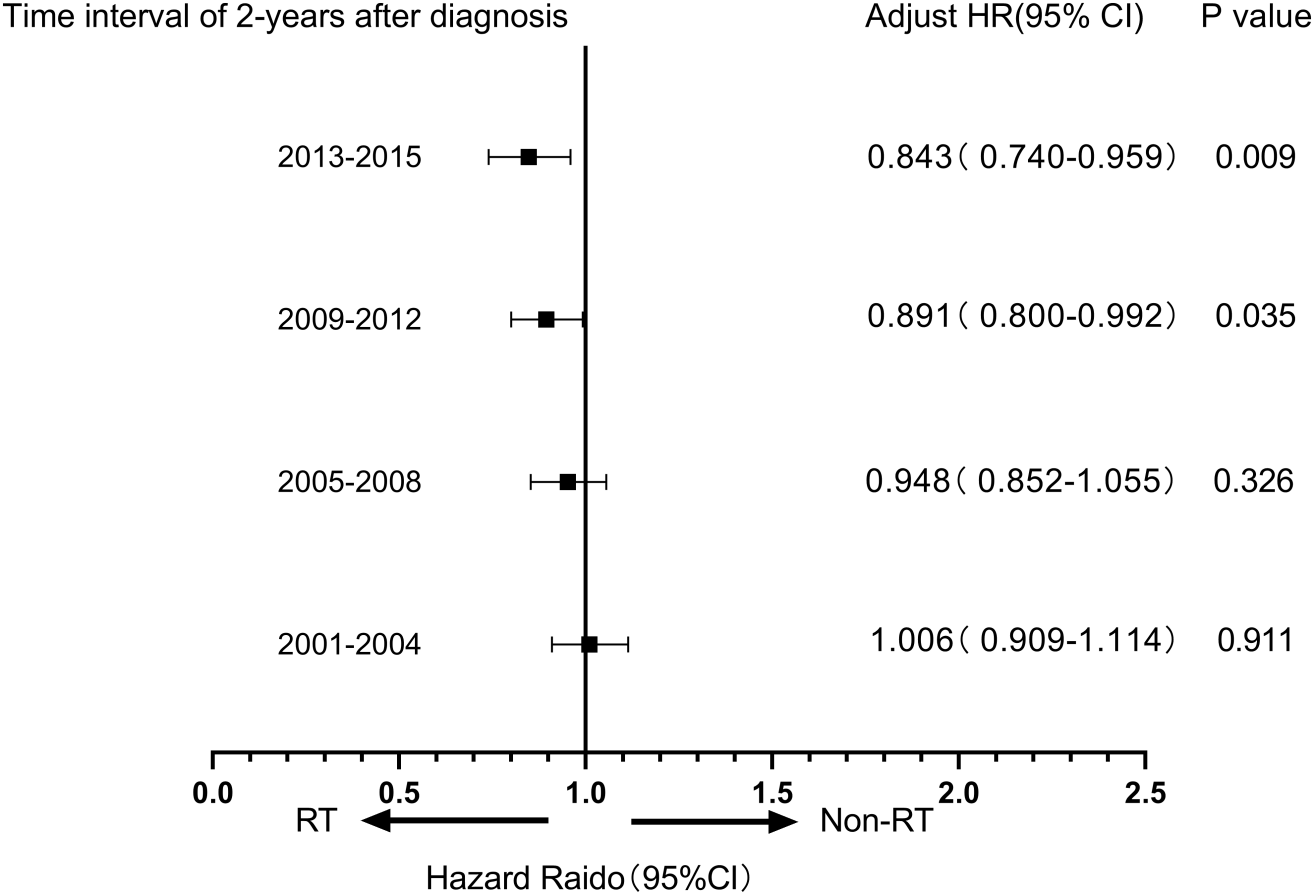
Hazard radio of RT versus non‐RT with 2 years of CVD‐specific death in patients with NSCLC at four consecutive time intervals

The analysis revealed that RT played a protective role for CVD‐specific death within 2 years after their cancer diagnosis. Consequently, patients were subdivided into groups based on year of diagnosis (2001–2004, 2005–2008, 2009–2012, and 2013–2015). COX regression data showed that radiotherapy improved highestly over time (Table 2). Figure 4 presented the change in RT as a prognostic factor over years. No difference was observed between the two groups in the interval between 2001–2004 and 2005–2008. However, the risk attenuated to become a protective factor from diagnosis years between 2009 and 2012 (HR = 0.891, 95% CI 0.800–0.992; *p* = 0.035) and especially 2013 to 2015 (HR = 0.843, 95% CI 0.740–0.959; *p* = 0.009). The result suggested that radiotherapy became increasingly protective over time. In addition, poorly differentiated grade (Grades III and IV) and RT were associated with an increased risk of CVD‐specific death at all intervals.

**TABLE 2:** Multivariate analysis of CVD‐specific death hazard for patients with NSCLC at four consecutive time intervals

	2001–2004				2005–2008				2009–2012				2013–2015			
Varibles	Univariate	Munivariate			Univariate	Munivariate			Univariate	Munivariate			Univariate	Munivariate		
	*p*	HR	95% CI	*p*	*p*	HR	95% CI	*p*	*p*	HR	95% CI	*p*	*p*	HR	95% CI	*p*
					0.011			0.010								
Race	0.538	Reference				Reference			0.331	Reference			0.243	Reference		
White					0.545	1.051	0.895–1.233	0.547								
Black	0.468				0.003	1.444	1.134–1.838	0.003	0.541				0.012			
Others	0.781								0.421				0.857			
Sex	0.561				0.241				0.333				0.206			
Female		Reference				Reference				Reference				Reference		
Male	0.561				0.241				0.333				0.206			
Age, years	0.331				0.335				0.665				0.175			
<65		Reference				Reference				Reference				Reference		
≥65	0.331				0.335				0.665				0.175			
Grade	0.005			0.006	0.002			0.001	0.000			0.000	0.018			0.006
I and II		Reference				Reference				Reference				Reference		
III and IV	0.005	1.148	1.040–1.268	0.006	0.002	1.202	1.084–1.332	0.000	0.000	1.236	1.114–1.371	0.000	0.018	1.191	1.051–1.351	0.006
Laterality	0.018			0.023	0.767				0.925				0.153			
Left		Reference				Reference				Reference				Reference		
Right	0.058	1.098	0.994–1.212	0.064	0.843				0.607				0.260			
Others	0.054	1.343	0.972–1.855	0.074	0.061				0.040				0.130			
Tumor histology	0.158				0.291				0.974				0.818			
Squamous and transitional cell		Reference				Reference				Reference				Reference		
Adenocarcinoma:	0.053				0.938				0.337				0.738			
Others	0.049				0.215				0.010				0.052			
Radiotherapy	0.911				0.326				0.073			0.000	0.026			0.009
No		Reference				Reference				Reference				Reference		
Yes	0.911				0.326				0.108				0.026	0.843	0.740–0.959	0.009
Chemotherapy	0.533				0.005			0.001	0.108			0.035	0.428			
No		Reference				Reference				Reference				Reference		
Yes	0.533				0.005	0.826	0.740–0.924	0.001	0.108	0.891	0.800–0.992	0.035	0.428			

Similarly, we did not obtain similar results for patients with small cell lung cancer in the SEER database (Appendix S4).

## 4 DISCUSSION

We showed that CVD‐specific mortality increased, although overall mortality from NSCLC declined in SEER registries. Lung cancer resulted in the highest mortality among all causes of death, followed by CVD. The increase in CVD‐specific death may be influenced by treatment and survival benefit, where oncotherapy may play a major role. A study published in Nature Communication has shown that cancer patients suffered from cardiovascular disease, which was generally a side effect caused by oncology. The CVD‐specific mortality rate was 10.61 per 10,000 person‐years, while the standardized mortality rate (SMR) for fatal heart disease was up to 2.24.^6^ A previous study has revealed that 12.9% of patients with lung cancer suffered from congestive heart failure.^7^ The survival of patients with NSCLC was affected by CVD according to the tumor stage and treatment, particularly multiple co‐existing CVD. Finally, some patients may acquire CVD after tumor diagnosis, even if they do not have any CVD risk factors. Improvements in treatment and increased survival times have made it more important to focus on the long‐term effects of cancer therapy and comorbidities, however, guidelines for lung cancer treatment generally do not make concrete recommendations for patients based on comorbidities. This condition should be considered when improving the cancer treatment selection process. Radiotherapy in patients with lung cancer is known to cause radiation‐induced heart disease.^8^ The biophysical effects of RT are selective not only for tumor cells, but also for the surrounding organs and tissues, which leads to adverse reactions. Moreover, targeted therapy may lead to hypertension and myocarditis of immunotherapy, or combined therapy may aggravate adverse reactions. The survival benefit for patients treated with combined therapy has been demonstrated, but it may increase CVD‐specific death. In turn, CVD may affect cancer risk, evolution, and treatment choice.^9^, ^10^, ^11^ Additionally, cardiotoxicity and CVD suffered from common risk factors (smoking and hyperlipidemia). And target organ damage (heart and lung) may affect cardiovascular mortality as longer survival time increases.

The effect of RT in CVD‐specific death increased continuously over the third years. Such a trend might suggest that long‐term cardiotoxicity emerges as a negative effect of radiotherapy in 3 years after receiving RT. Of particular relevance to our results was a report suggesting that patients with lung cancer who have been exposed to radiation may be at increased risk of MACE, and the mean radiation dose delivered to the heart (mean cardiac dose) was associated with a significantly increased risk of MACE. After a median follow‐up of 20.4 months in 748 patients, 77 patients developed ≥1 MACE (2‐year cumulative incidence.^12^ A study by Guberina et al. has reported that the average cardiac radiation dose (Heart V5) was not associated with survival.^13^ Otherwise, the incidence of MACE increased when the average cardiac dose received was >10 Gy in patients without a history of CVD,^12^ which supports the need for early recognition and treatment of cardiovascular events and more stringent avoidance of high cardiac radiotherapy dose. RT improves the outcomes of NSCLC treatment. Approximately half of all patients with cancer receive RT during their care.^14^ Technological advances have dramatically improved radiation therapy, but the long‐term effects are still significant and cannot be ignored. Radiotherapy should be carefully considered when patients have risk factors for cardiovascular disease. We can try to minimize the impact on the heart by adjusting radiation field and radiotherapy dose parameters. Most lung cancer clinical trials have excluded patients with comorbidities to avoid obscuration of cancer treatment effects by non‐lung cancer‐specific conditions. Clinical symptoms of patients are mostly considered for tumor disease presence instead of comorbidities or therapeutic toxicity. In our opinion, there was a lack of consistency in the clinical use of records of patients with pre‐existing heart disease prior to therapy, mean cardiac dose, study endpoints used, and treatment of patients with comorbidities in current studies. The American Society of Clinical Oncology and the European Society of Cardiology has published consensus and guidelines for the management of cardiotoxicity related to tumor treatment and proposed pathways for monitoring and managing potentially cardiotoxicity treatments, including risk‐stratified assessment and cardiotoxicity monitoring procedures.^15^, ^16^ The identification and management of cardiac toxicity in patients may be secondary to improvements in treatment planning and delivery of thoracic radiotherapy.

Our study showed that RT had a short‐term protective effect within 2 years. Heart may have self‐compensation mechanism in the short‐term effect to avoid the impact of radiotherapy. Previous studies have suggested that radiation caused progressive renin–angiotensin–aldosterone system (RAAS)‐driven remodeling of heart.^17^ The increased sympathetic stimulation served to compensate for subclinical myocardial injury to prevent cardiac fibrosis and protect, but sympathetic overactivation led to end‐stage symptoms.^18^, ^19^ Therefore, the heart may be in the repair stage of radiation injury within 2 years, while RT played a major role in treating cancer. The HR of RT was 1.6 from 2001 to 2004, and then gradually decreased to 0.843 from 2013 to 2015. We speculated that our results may represent improvements in radiotherapy techniques that minimize heart irradiation. Technical improvements in the application of thoracic radiation therapy for lung cancer, particularly with the integration of computed tomography (CT)‐based treatment planning, have greatly improved our ability to identify tumors and to delineate normal tissues. The early 1990s were associated with the combination of a linear accelerator with CT imaging, resulting in three‐dimensional conformal radiotherapy. Image‐guided radiotherapy assisted in patient localization throughout and reduced heart further away from the high‐dose region.^20^, ^21^ Moreover, intensity‐modulated radiotherapy may reduce the combined cardiopulmonary dose and improve the therapeutic ratio.^22^ Furthermore, passive scattering proton therapy may be beneficial to the heart because it exposes less heart tissue between 5 and 80 Gy.^23^ Finally, clinicians pay attention to the heart substructure through a map description of thoracic radiotherapy.^24^ Improvements in these various components of radiation therapy have resulted in an improved ability to deliver safe radiation doses to primary tumors. Advances in radiotherapy techniques have reduced cardiotoxicity associated with radiotherapy. We did not obtain similar results for small cell lung cancer, which may be due to the lack of progress in the treatment of small cell lung cancer.

Data on specific radiotherapy techniques and patient comorbidities (dose, segmentation, ray energy, etc.) were not available in the SEER database and were not included in this analysis. Detailed volume information and individual doses would have allowed us to identify the specific techniques that increase CVD‐specific death. Comorbid conditions present at NSCLC diagnosis are likely associated both with the treatment received and heart disease mortality. However, we do not have detailed data on this variable.

Although the technology has improved, the cardiotoxicity of chest radiotherapy was inevitable, and studies on RT‐induced cardiotoxicity and its impact on mortality are still limited. We found the cardiotoxicity about the key point time in time, and it lasted for a long time. It turns out that RT is protective at 2 years. We suggest intervention in this cardiotoxicity at as early as 2 years. Given the improvement in NSCLC outcomes after radiotherapy and the occurrence of clinically important cardiac events, strategies to avoid and manage cardiac toxicity are needed. Preventive measures may include the use of beta‐blockers and statins, which may prevent adverse cardiac remodeling and significantly reduce cardiac events.^25^, ^26^

## 5 CONCLUSION

Although the mortality from NSCLC decreased, radiotherapy‐related CVD‐specific mortality cannot be ignored. In the long‐term over 3 years, RT significantly promoted CVD‐specific death. However, RT turned to play a protective role in the short‐term within 2 years. We need to comprehensively consider the dual effects of radiotherapy on the side effects on the heart in clinical practice.

## AUTHOR CONTRIBUTIONS

Jinming Yu: Conceptualization and design, financial support, administrative support, provision of study materials and/or patients, collection and assembly of data, and data analysis and interpretation. Dawei Chen: Conceptualization and design, financial support, and data analysis and interpretation. You Mo: Provision of study materials and/or patients, and collection and assembly of data. Baoqing Tian: Provision of study materials and/or patients and collection and assembly of data. Meng Wu: Assembly of data. Minxin Chen: Data analysis and interpretation.

## FUNDING INFORMATION

Dawei Chen has received grants from National Natural Science Foundation of China (82172676), Science Foundation of Shandong (ZR2020LZL016, ZR2021YQ52), Foundation of Bethune Charitable(2021434953) and Young Elite Scientist Sponsorship Program By Cast (NO. YESS20210137). Jinming Yu has received grants from the foundation of National Natural Science Foundation of China (81627901, 81972863 and 82030082).

## CONFLICT OF INTEREST

The authors declare that they have no competing interests.

## ETHICAL APPROVAL STATEMENT

Because the SEER database is publicly accessible worldwide, therefore, we did not provide the approval of an institutional review board in the current study. But the authors signed the SEER database agreement and got the license to access SEER information (accession username: 18157‐Nov 2019).

## Supporting information


Appendix S1

## Data Availability

The data is available in the SEER program.
