# Supplementary material for: Dual effect of radiotherapy related concomitant cardiovascular diseases in non‐small cell lung cancer
Source: Cancer Med. 2022 Jun 26;12(2):1025–34. doi: 10.1002/cam4.4948 (PMC9883436; doi:10.1002/cam4.4948)
Supplement: Supplementary file 1 — Appendix S1 [file CAM4-12-1025-s001.zip › cam44948-sup-0002-FigureS2.docx]

| S2. Multivariate Analysis of Cardiac specific death Hazard for Patients With Nonsmall Cell Lung Cancer | | | | | | | | | | | | | | | | | | | | | | | |
| --- | --- | --- | --- | --- | --- | --- | --- | --- | --- | --- | --- | --- | --- | --- | --- | --- | --- | --- | --- | --- | --- | --- | --- |
|  | 2-years | | | | | | |  | 3-years | | | | | | |  | 4-years | | | | | | |
| Varibles | Univariate | | |  | Munivariate | | |  | Univariate |  |  |  | Munivariate | | |  | Univariate | | |  | Munivariate | | |
|  | HR | 95% CI | *P* |  | HR | 95% CI | *P* |  | HR | 95% CI | *P* |  | HR | 95% CI | *P* |  | HR | 95% CI | *P* |  | HR | 95% CI | *P* |
|  |  | | |  |  |  |  |  |  |  |  |  |  |  |  |  |  |  |  |  |  |  |  |
| Race |  |  | 0.009 |  |  |  | 0.015 |  |  |  | 0.000 |  |  |  | 0.000 |  |  |  | 0.000 |  |  |  | 0.000 |
| White | Reference |  |  |  | Reference |  |  |  | Reference |  |  |  | Reference |  |  |  | Reference |  |  |  |  |  |  |
| Black | 1.084 | 1.000-1.175 | 0.049 |  | 1.081 | 0.997-1.172 | 0.059 |  | 1.101 | 1.024-1.185 | 0.010 |  | 1.1 | 1.023-1.184 | 0.010 |  | 1.145 | 1.068-1.227 | 0.000 |  | 1.136 | 1.060-1.218 | 0.000 |
| Others | 1.127 | 0.997-1.275 | 0.057 |  | 1.116 | 0.987-1.263 | 0.081 |  | 1.19 | 1.062-1.333 | 0.003 |  | 1.189 | 1.61-1.332 | 0.003 |  | 1.124 | 1.010-1.251 | 0.032 |  | 1.121 | 1.007-1.247 | 0.036 |
| Sex |  |  | 0.051 |  |  |  | 0.053 |  |  |  | 0.000 |  |  |  | 0.000 |  |  |  | 0.000 |  |  |  | 0.000 |
| Male | Reference | | |  | Reference |  |  |  | Reference |  |  |  | Reference | | |  | Reference |  |  |  | Reference |  |  |
| Female | 0.947 | 0.896-1.000 | 0.051 |  | 0.947 | 0.896-1.000 | 0.050 |  | 0.911 | 0.867-0.957 | 0.000 |  | 0.914 | 0.870-0.960 | 0.000 |  | 0.904 | 0.863-0.947 | 0.000 |  | 0.909 | 0.868-0.952 | 0.000 |
| Age,years |  |  | 0.174 |  |  |  |  |  |  |  | 0.429 |  |  |  |  |  |  |  | 0.408 |  |  |  |  |
| <65 |  | | |  |  |  |  |  |  |  |  |  |  |  |  |  |  |  |  |  |  |  |  |
| ≥65 |  |  |  |  |  |  |  |  |  |  |  |  |  |  |  |  |  |  |  |  |  |  |  |
| Grade |  |  | 0.000 |  |  |  | 0.000 |  |  |  | 0.000 |  |  |  | 0.000 |  |  |  | 0.000 |  |  |  | 0.000 |
| I and II | Reference |  |  |  | Reference |  |  |  | Reference |  |  |  | Reference |  |  |  | Reference |  |  |  | Reference |  |  |
| III and IV | 1.184 | 1.123-1.248 | 0.000 |  | 1.191 | 1.129-1.256 | 0.000 |  | 1.149 | 1.096-1.205 | 0.000 |  | 1.142 | 1.089-1.198 | 0.000 |  | 1.161 | 1.110-1.214 | 0.000 |  | 1.136 | 1.086-1.189 | 0.000 |
| Laterality |  |  | 0.045 |  |  |  | 0.034 |  |  |  | 0.001 |  |  |  | 0.001 |  |  |  | 0.000 |  |  |  | 0.000 |
| Left | Reference |  |  |  | Reference |  |  |  | Reference |  |  |  | Reference |  |  |  | Reference |  |  |  | Reference |  |  |
| Right | 1.03 | 0.976-1.086 | 0.283 |  | 1.034 | 0.980-1.091 | 0.225 |  | 1.058 | 1.009-1.111 | 0.021 |  | 1.063 | 1.013-1.115 | 0.013 |  | 1.071 | 1.024-1.120 | 0.003 |  | 1.077 | 1.030-1.127 | 0.001 |
| Others | 1.504 | 1.205-1.877 | 0.000 |  | 1.188 | 1.192-1.858 | 0.000 |  | 1.532 | 1.243-1.887 | 0.000 |  | 1.511 | 1.226-1.862 | 0.000 |  | 1.787 | 1.454-2.196 | 0.000 |  | 1.733 | 1.409-2.130 | 0.000 |
| Tumor histology |  |  | 0.827 |  |  |  |  |  |  |  | 0.814 |  |  |  |  |  |  |  | 0.418 |  |  |  |  |
| squamous and transitional cell |  |  |  |  |  |  |  |  |  |  |  |  |  |  |  |  |  |  |  |  |  |  |  |
| Adenocarcinoma: |  |  |  |  |  |  |  |  |  |  |  |  |  |  |  |  |  |  |  |  |  |  |  |
| Others |  |  |  |  |  |  |  |  |  |  |  |  |  |  |  |  |  |  |  |  |  |  |  |
| Radiation |  |  | 0.022 |  |  |  | 0.003 |  |  |  | 0.267 |  |  |  |  |  |  |  | 0.000 |  |  |  | 0.006 |
| No | Reference |  |  |  |  |  |  |  |  |  |  |  |  |  |  |  | Reference |  |  |  | Reference |  |  |
| Yes | 0.938 | 0.888-0.991 | 0.022 |  | 0.917 | 0.867-0.969 | 0.002 |  |  |  |  |  |  |  |  |  | 1.112 | 1.061-1.165 | 0.000 |  | 1.075 | 1.022-1.132 | 0.005 |
| Chemothreapy |  |  | 0.453 |  |  |  |  |  |  |  | 0.662 |  |  |  |  |  |  |  | 0.000 |  |  |  | 0.083 |
| No |  |  |  |  |  |  |  |  |  |  |  |  |  |  |  |  | Reference |  |  |  | Reference |  |  |
| Yes |  |  |  |  |  |  |  |  |  |  |  |  |  |  |  |  | 1.109 | 1.055-1.166 | 0.000 |  | 1.045 | 0.990-1.103 | 0.083 |

Continued

| Multivariate Analysis of Cardiac specific death Hazard for Patients With Nonsmall Cell Lung Cancer | | | | | | | | | | | | | | | |
| --- | --- | --- | --- | --- | --- | --- | --- | --- | --- | --- | --- | --- | --- | --- | --- |
|  | 5-years | | | | | | |  | 6-years | | | | | | |
| Varibles | Univariate | | |  | Munivariate | | |  | Univariate | | |  | Munivariate | | |
|  | HR | 95% CI | *P* |  | HR | 95% CI | *P* |  | HR | 95% CI | *P* |  | HR | 95% CI | *P* |
|  |  |  |  |  |  |  |  |  |  |  |  |  |  |  |  |
| Race |  |  | 0.000 |  |  |  | 0.000 |  |  |  | 0.000 |  |  |  | 0.000 |
| White | Reference |  |  |  | Reference |  |  |  | Reference |  |  |  | Reference |  |  |
| Black | 1.138 | 1.064-1.217 | 0.000 |  | 1.13 | 1.059-1.211 | 0.000 |  | 1.169 | 1.096-1.248 | 0.000 |  | 1.161 | 1.088-1.239 | 0.000 |
| Others | 1.133 | 1.023-1.255 | 0.017 |  | 1.116 | 1.007-1.237 | 0.036 |  | 1.14 | 1.032-1.259 | 0.010 |  | 1.13 | 1.023-1.248 | 0.016 |
| Sex |  |  | 0.000 |  |  |  | 0.000 |  |  |  | 0.000 |  |  |  | 0.000 |
| Male | Reference |  |  |  | Reference |  |  |  | Reference |  |  |  |  |  |  |
| Female | 0.888 | 0.850-0.928 | 0.000 |  | 0.896 | 0.858-0.937 | 0.000 |  | 0.888 | 0.851-0.926 | 0.000 |  |  |  |  |
| Age,years |  |  | 0.567 |  |  |  |  |  |  |  | 0.860 |  |  |  |  |
| <65 |  |  |  |  |  |  |  |  |  |  |  |  |  |  |  |
| ≥65 |  |  |  |  |  |  |  |  |  |  |  |  |  |  |  |
| Grade |  |  | 0.000 |  |  |  | 0.000 |  |  |  | 0.000 |  |  |  | 0.000 |
| I and II | Reference |  |  |  | Reference |  |  |  | Reference |  |  |  | Reference |  |  |
| III and IV | 1.145 | 1.097-1.195 | 0.000 |  | 1.107 | 1.060-1.156 | 0.000 |  | 1.156 | 1.110-1.204 | 0.000 |  | 1.11 | 1.065-1.158 | 0.000 |
| Laterality |  |  | 0.017 |  |  |  | 0.006 |  |  |  | 0.001 |  |  |  | 0.000 |
| Left | Reference |  |  |  | Reference |  |  |  | Reference |  |  |  | Reference |  |  |
| Right | 1.025 | 0.982-1.070 | 0.264 |  | 1.033 | 0.990-1.079 | 0.136 |  | 1.049 | 1.006-1.093 | 0.024 |  | 1.066 | 1.023-1.112 | 0.002 |
| Others | 1.827 | 1.490-2.240 | 0.000 |  | 1.838 | 1.498-2.254 | 0.000 |  | 1.809 | 1.480-2.210 | 0.000 |  | 1.716 | 1.404-2.098 | 0.000 |
| Tumor histology |  |  | 0.569 |  |  |  |  |  |  |  | 0.141 |  |  |  |  |
| squamous and transitional cell |  |  |  |  |  |  |  |  |  |  |  |  |  |  |  |
| Adenocarcinoma: |  |  |  |  |  |  |  |  |  |  |  |  |  |  |  |
| Others |  |  |  |  |  |  |  |  |  |  |  |  |  |  |  |
| Radiation |  |  | 0.000 |  |  |  | 0.000 |  |  |  | 0.000 |  |  |  | 0.000 |
| No | Reference |  |  |  | Reference |  |  |  | Reference |  |  |  | Reference |  |  |
| Yes | 1.203 | 1.150-1.259 | 0.000 |  | 1.164 | 1.108-1.223 | 0.000 |  | 1.273 | 1.218-1.331 | 0.000 |  | 1.219 | 1.162-1.280 | 0.000 |
| Chemothreapy |  |  | 0.000 |  |  |  | 0.070 |  |  |  | 0.000 |  |  |  | 0.015 |
| No | Reference |  |  |  | Reference |  |  |  | Reference |  |  |  | Reference |  |  |
| Yes | 1.14 | 1.086-1.195 | 0.000 |  | 1.046 | 0.992-1.102 | 0.095 |  | 1.181 | 1.127-1.237 | 0.000 |  | 1.062 | 1.009-1.118 | 0.022 |
