# Supplementary figures and images for: Dual effect of radiotherapy related concomitant cardiovascular diseases in non‐small cell lung cancer
Source: Cancer Med. 2022 Jun 26;12(2):1025–34. doi: 10.1002/cam4.4948 (PMC9883436; doi:10.1002/cam4.4948)

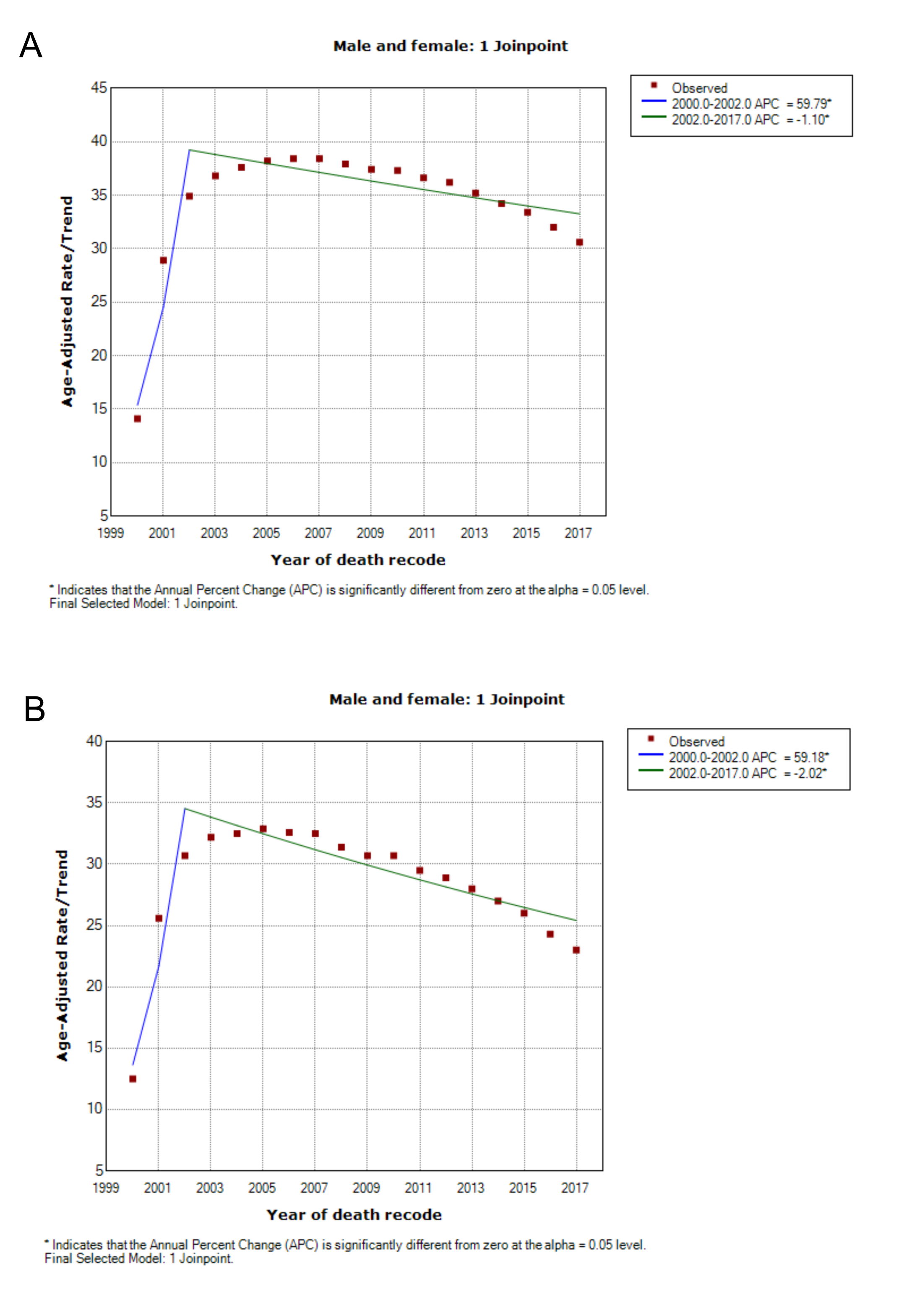

Supplement: Supplementary file 1 — Appendix S1 [file CAM4-12-1025-s001.zip › cam44948-sup-0001-FigureS1.png]

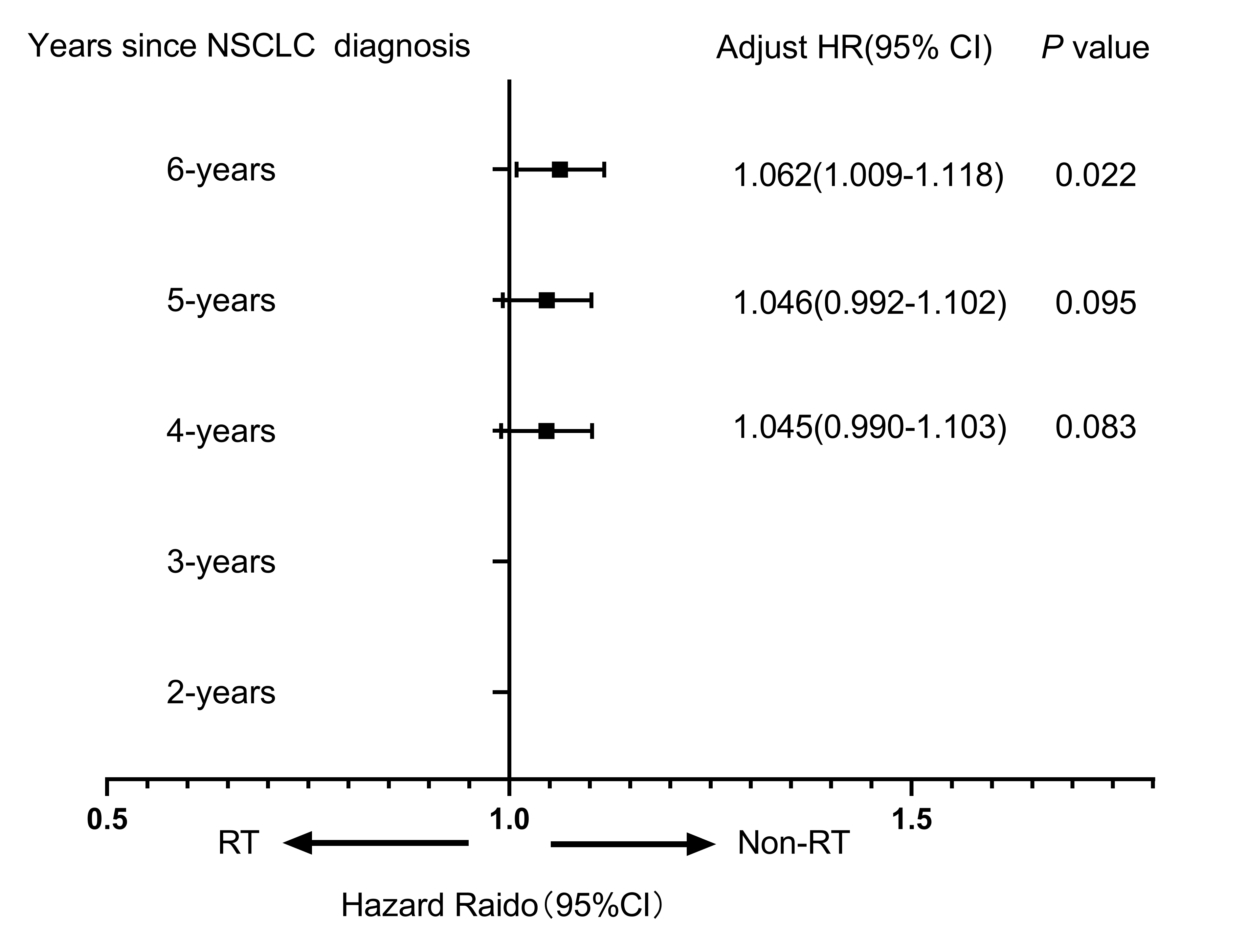

Supplement: Supplementary file 1 — Appendix S1 [file CAM4-12-1025-s001.zip › cam44948-sup-0003-FigureS3.jpg]

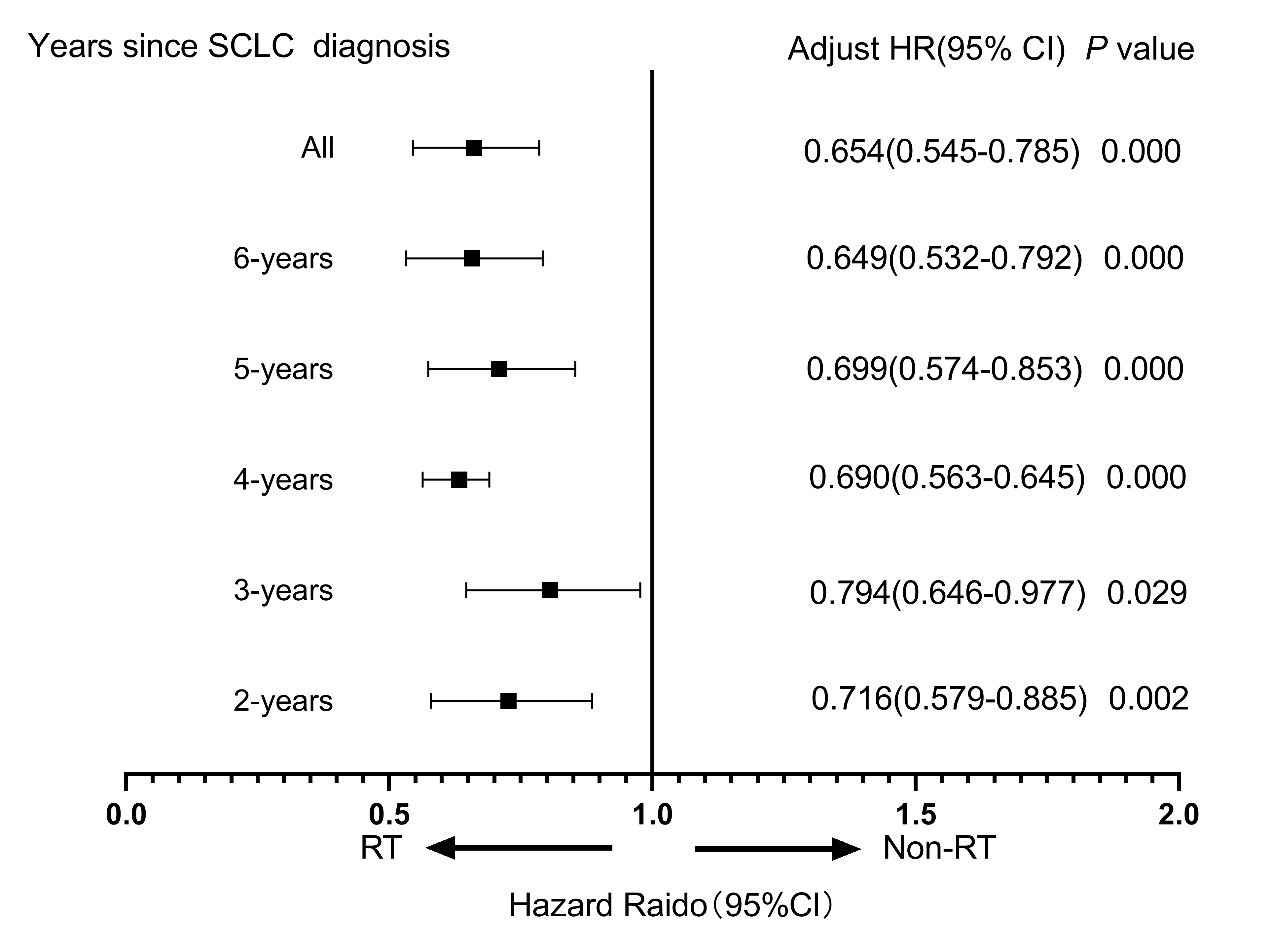

Supplement: Supplementary file 1 — Appendix S1 [file CAM4-12-1025-s001.zip › cam44948-sup-0004-FigureS4.tif]
